# Cost Comparison of Prostatic Artery Embolization Between In-Hospital and Outpatient-Based Lab Settings

**DOI:** 10.7759/cureus.67433

**Published:** 2024-08-21

**Authors:** Lucas R Cusumano, Johann S Rink, Tyler Callese, Cleo K Maehara, Sipan Mathevosian, Matthew Quirk, Adam Plotnik, Justin P McWilliams

**Affiliations:** 1 Department of Radiological Sciences, David Geffen School of Medicine at University of California, Los Angeles, USA; 2 Department of Clinical Radiology and Nuclear Medicine, Mannheim University Medical Centre, Mannheim, DEU; 3 Department of Radiological Sciences, David Geffen School of Medicine at University of California, Los Angeles, Los Angeles, USA

**Keywords:** outpatient-based lab, lower urinary tract symptoms, benign prostatic hyperplasia (bph), time-driven activity-based costing, prostatic artery embolization

## Abstract

Purpose

This study aimed to determine the costs associated with prostatic artery embolization (PAE) performed in hospital and outpatient-based lab (OBL) settings.

Methods

Procedures were performed in similarly equipped procedure suites located within a tertiary hospital or OBL. Time-driven activity-based costing (TDABC) was utilized to calculate procedural costs incurred by the institution. Process maps were created describing personnel, space, equipment, and materials. The time duration of each procedural step was recorded independently by a nurse caring for the patient at the time of the procedure, and mean values were included in our model. Using institutional and publicly available financial data, costs, and capacity cost rates were determined.

Results

Thirty-seven PAE procedures met inclusion criteria with a mean patient age of 70.4 (+/- 6.7) years and a mean prostate gland size of 129.7 (+/-56.4) cc. Twenty-six procedures were performed within the hospital setting, and 11 procedures were performed within the OBL. Reduction in International Prostate Symptom Score (IPSS) was not significantly different following hospital and OBL procedures (57.2% vs. 82.4%, P = 0.0796). Mean procedural time was not significantly different between the hospital and OBL settings (136.6 vs. 147.3 minutes, P = 0.1893). However, the duration between admission and discharge was significantly longer for procedures performed in a hospital (468.8 vs. 325.4 minutes, P <0.0001). Total costs for hospital-based procedures were marginally higher ($3,858.28 vs. $3,642.67).

Conclusion

Total PAE cost was similar between the hospital and OBL settings. However, longer periprocedural times for hospital-based procedures and differences in reimbursement may favor the performance of PAE in an OBL setting.

## Introduction

Benign prostatic hyperplasia (BPH) is a medical condition affecting most men over 50 years old and is associated with bladder outlet obstruction (BOO) and lower urinary tract symptoms (LUTS) [1, 2]. This may impede everyday activities and cause complications such as urinary retention, infections, bladder stones, and renal insufficiency, leading to a significant decrease in health-related quality of life [3, 4]. The condition creates a healthcare burden, which was estimated to be around $3.9 billion annually in the United States of America (USA) in 2010 [4].

If medical management fails to control LUTS, surgical treatment is recommended. Transurethral resection of the prostate (TURP) is considered the gold standard of treatment, as it is known to be effective and durable in symptom control [5]. However, possible side effects of TURP, such as sexual dysfunction, incontinence, and the risk of developing strictures, have led to a search for potential alternative treatment strategies [6,7].

Prostatic artery embolization (PAE) has proven safe and effective for BPH symptom control [8-10] and may also be performed as an outpatient procedure, which typically requires only a few hours of recovery compared to TURP, which often necessitates a one-day hospitalization period [9,11]. Recent economic analyses from the USA [12,13], Canada [14], Switzerland [15], and Spain [16] have also compared direct healthcare costs arising from TURP and PAE, consistently finding PAE to be associated with lower costs.

As PAE is safe and cost-effective when performed as an outpatient procedure, further scientific attention seems to be justified in optimizing the treatment setting. It can be performed either in interventional radiology (IR) departments of larger hospitals or in an outpatient-based lab (OBL) specializing in IR procedures. This might not only maximize patient comfort but also enable streamlining of patient preparation and treatment processes to reduce costs. Little is known about possible cost differences from performing PAE in these different settings.

Time-driven activity-based costing (TDABC) is a microcosting method that generates procedural costs by using estimates of the per unit cost of resources (e.g., personnel, space, equipment, and materials) and the time and quantity of each resource required over a given patient care cycle. This technique has been described as a promising method capable of improving healthcare performance by helping institutions improve the allocation of costs for supplies, hardware, infrastructure, and professional charges with the ultimate goal of increasing value in healthcare [17].

The purpose of this study was to utilize TDABC to compare the costs of performing outpatient PAE in the IR department of a large academic-level hospital versus an OBL.

## Materials and methods

Time-driven activity-based costing analysis

Institutional review board approval was obtained from the University of California, Los Angeles Institutional Review Board (approval number: 18-001455), which provided ethical approval of the study design and waiver of patient informed consent. This TDABC model was developed in accordance with guidelines set forth by the TDABC for Healthcare Consortium and previously reported methods [18-20].

Identify the medical condition and patient care cycle

Patients who underwent PAE for treatment of BPH between January 2021 and December 2021 were retrospectively identified and reviewed. International Prostate Symptom Score (IPSS) was calculated at the pre-procedure clinic visit and first post-procedural clinic follow-up at approximately three months following PAE. Pre-procedure and post-procedure IPSS scores were reported for 18 patients in the hospital setting cohort and five patients in the OBL cohort. All procedures were performed in similarly equipped IR suites located in either a tertiary hospital or OBL. All procedures were performed with right common femoral artery access. Exclusion criteria included history of prior PAE and absence of resident physician assistance with procedures. The process maps of TDABC maps are defined by a single care cycle, defined here as beginning with the patient's admission to the preoperative unit and ending with discharge from the recovery unit. 

Process map development

Detailed process maps, including the timing of each step and required resources (personnel, space, equipment, and materials), were developed for PAEs performed in the hospital and OBL settings. Process maps were designed for a web-based TDABC software application, TDABC Designer (University of California, Los Angeles (UCLA) Department of Radiology, Los Angeles, CA), which integrates data from several sources, including radiology, finance, and human resources [21]. 

Resource identification and time estimates

A mixed methods approach facilitated internal data validation across multiple sources [20, 22]. Sources included the electronic medical record (EMR), institutional and departmental financial data, pharmacy costs data, and direct observation.

The EMR encounters, operative reports, and nursing notes were reviewed for operative times, inventory used, and charge data. Mean times for each step were included in each process map. A multidisciplinary team of stakeholders was identified and interviewed, including interventional radiologists, nursing, and IR technologists, and administrative financial managers. Interviews were unstructured and focused on identifying process steps and resource utilization.

Establishing resource costs

Utilizing TDABC Designer, resource costs were generated and organized into four cost domains: personnel, space, equipment, and materials. Personnel included the attending physician, resident physician, IR technologist, and nurse. Personnel costs were identified using publicly available institutional salary data [23]. Space costs refer to the costs to acquire, maintain, and utilize the pre-procedure, IR suite, and post-procedure areas within the hospital or OBL. Equipment costs refer to apparatus related to image guidance, such as fluoroscopy equipment. Materials costs refer to the additional items utilized during patient care and treatment, including catheters, wires, medications, embolic agents, and patient supplies. Space costs, equipment costs (including capital costs), and material costs were derived from multiple institutional sources and integrated into the TDABC Designer application. The integrated database included fiscal year 2021 data.

The PAE technique

All procedures were performed by one of three operators with five to 15 years of experience. All patients received one dose of intravenous ciprofloxacin 400 mg for antibiotic prophylaxis. All procedures were performed under moderate sedation (intravenous midazolam and fentanyl) via right common femoral artery access. Internal iliac angiography in an ipsilateral oblique projection was performed to identify the right and left prostatic arteries using a 5-F Cobra 2 (Cook Medical, Bloomington, IN) catheter for the contralateral side, and a Simmons 1 (Cook Medical) catheter for the ipsilateral side. All arteries supplying the prostate were catheterized using a 2.0-F Progreat Alpha (Terumo Interventional Systems, Somerset, NJ) microcatheter and 0.014-inch Synchro Soft (Stryker Neurovascular, Fremont, CA) microguidewire. Using 300-500 μm Embospheres (Merit Medical Systems, Inc., South Jordan, UT) microspheres, bilateral PAE was then performed to stasis. Patients were discharged home the same day following recovery with clinic follow-up at approximately three and 12 months after the procedure.

Data analysis

Capacity cost rates (CCR) describe the cost per minute of a given resource (i.e., personnel, space, and equipment). The CCR ($USD/min) was calculated for each resource by dividing resource costs ($USD/year) by its practical capacity (minutes/year) [24]. Costs were calculated by multiplying the CCR by time for each step in the process map. 

Costs were summed for each step in the individual process maps to provide the total cost for each respective treatment setting. Total costs were then further divided into personnel, space, equipment, and materials costs. 

Statistical analyses were performed using GraphPad Prism, version 9.2 (GraphPad Software, La Jolla, CA). Continuous data are expressed as mean values with standard deviation. Comparisons between groups were made using the Mann-Whitney analysis. Two-tailed p-values <0.05 were considered statistically significant.

## Results

Prostatic artery embolization procedures and patient characteristics

A total of 37 PAE procedures were identified for analysis. Twenty-six PAE procedures were performed in the hospital setting. Eleven PAE procedures were performed within an OBL. We found no significant difference between patient characteristics, including age, history of prior prostate surgery, mean prostate gland size, and reduction in IPSS, as summarized in Table 1. 

**Table 1:** Patient characteristics Comparisons between groups were made using the Mann-Whitney analysis.

Characteristic	Hospital setting (N = 26)	Outpatient-based lab (N = 11)	P-value
Age (years)	69.50 (± 7.2)	72.36 (± 4.9)	0.2952
Prior prostate surgery	7 (26.9%)	2 (18.2%)	0.6946
Prostate cancer history	0 (0%)	1 (9.1%)	0.2873
Mean prostate gland size (grams)	113.0 (± 51.4)	121.6 (± 69.1)	0.4856
Reduction in International Prostate Symptom Score (IPSS)	57.21% (± 25.8%)	82.38% (± 7.1%)	0.0796

Process maps

**Figure 1:**
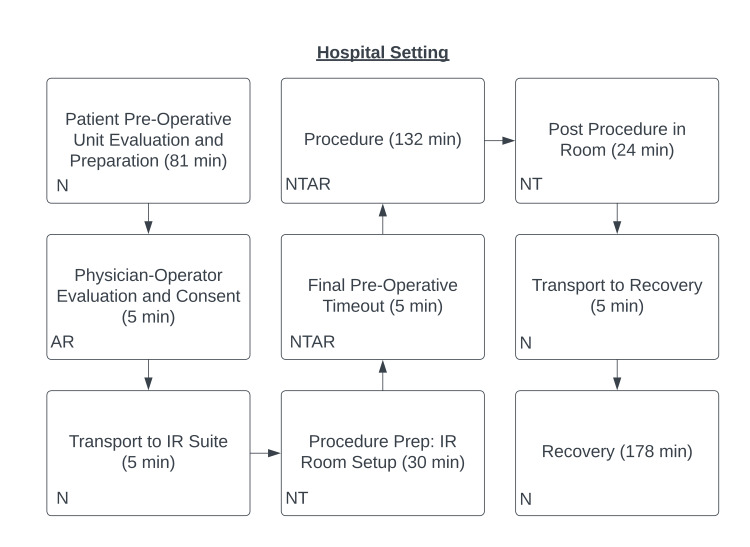
Time-driven activity-based costing (TDABC) process map for the tertiary hospital setting Personnel involved in patient care included an attending physician (A), a resident physician (R), an IR technologist (T), and a nurse (N).

**Figure 2:**
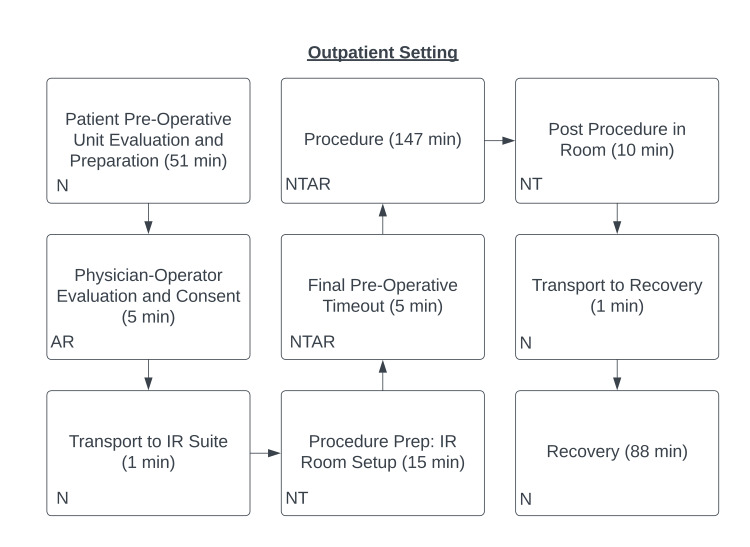
Time-driven activity-based costing (TDABC) process map for the outpatient lab setting Personnel involved in patient care included the attending physician (A), resident physician (R), interventional radiology (IR) technologist (T), and nurse (N).

The TDABC process maps were produced for the hospital and OBL setting (Figures 1-2).

The mean total hospital time was 468.8 ± 61.7 minutes compared to 325.4 ± 32.8 minutes within the OBL (P <0.0001). Mean procedure times in the hospital of 136.6 ± 18.4 minutes and 147.3 ± 22.8 minutes in the OBL were not significantly different (P = 0.1893). The mean pre-procedure time was significantly longer in the hospital setting (127.6 ± 43.5 minutes vs. 77.6 ± 19.8 minutes, P <0.0001). Mean post-procedure time was also significantly longer in the hospital setting (209.2 ± 56.9 minutes vs. 100.5 ± 27.0 minutes, P <0.0001).

Prostatic artery embolization costs

Cost data by activity step is listed in Table 2. Total PAE cost was determined to be $3,858.28 in the hospital setting and $3,642.67 with the OBL. Personnel costs accounted for 52.8% and 54.7% of total costs in the hospital and OBL settings, respectively. In the hospital setting, space costs were lower ($13.25 vs. $16.82) and equipment costs were higher ($128.16 vs. $118.77). There was no difference between material costs in either setting. Summary cost data are provided in Table 3.

**Table 2:** Time-driven activity-based costing (TDABC) by activity and setting The cost of each activity for a single patient was calculated based on the sum of personnel, materials, equipment, and space costs. Activity duration for each treatment setting was estimated by taking the mean time spent in each activity by study participants.

Activity	Hospital setting	Outpatient-based lab
Patient pre-operative unit evaluation and preparation	$145.49	$92.87
Physician-operator evaluation and consent	$32.48	$32.50
Transport to interventional radiology (IR) suite	$10.09	$2.03
Procedure preparation	$129.27	$65.03
Final pre-operative time out	$53.97	$54.10
Procedure	$3,029.01	$3,194.73
Post-procedure in room	$103.41	$43.35
Transport to recovery	$40.21	$2.03
Recovery	$314.35	$156.03
Total	$3,858.28	$3642.67

**Table 3:** Time-driven activity-based costing (TDABC) summary costs The cost of each resource for a single patient in a hospital setting and outpatient-based lab. Activity duration for each treatment setting was estimated by taking the mean time spent in each activity by study participants.

Costs	Hospital setting	Outpatient-based lab
Personnel	$2,110.10 (54.7%)	$1900.31 (52.2%)
Materials	$1,606.77 (41.6%)	$1606.77 (44.1%)
Equipment	$128.16 (3.3%)	$118.77 (3.3%)
Space	$13.25 (0.3%)	$16.82 (0.5%)
Total	$3,858.28	$3642.67

## Discussion

Treatment of BPH with LUTS with PAE is a viable option for patients desiring a minimally invasive approach [10]. The safety of PAE as a same-day procedure has been previously demonstrated, which positions it well as an ideal candidate to be performed in the OBL [25,26]. However, there is a paucity of data regarding the costs associated with PAE performed within the OBL, as prior studies have focused on the hospital setting [12-16]. This study builds on the literature to identify differences in cost drivers in PAE performed in the hospital and OBL settings. 

Accurate assessments of costs are essential in a value-based healthcare delivery system. However, estimating costs can be difficult due to the complex nature of medical procedures and the multiple stakeholders involved. Time-driven activity-based costing is a well-recognized method of systematically determining costs and has been utilized for several medical procedures [27]. Utilizing this methodology, we found a modestly increased total cost associated with PAE performed in the hospital compared to procedures performed in the OBL ($3,858.28 vs. $3,642.67). We attribute this to similarities in procedure times, personnel, and equipment/materials utilized. Our calculated cost was within the $1678-$6464 range reported by prior studies conducted in the USA [12,13]. Major differences in cost estimates between studies are predominantly due to variations in calculating personnel and institution-specific costs as well as how the care cycle was determined. For example, Bagla et al. did not include professional charges of the physicians involved in treatment [12]. However, personnel costs accounted for approximately 50% of procedure-related costs in the present study. One factor leading to our lower cost estimate compared to Rink et al. was their inclusion of postprocedural care after discharge from the recovery unit [13]. 

Despite overall similarities in costs between settings, the longer perioperative period in the hospital setting costs an additional $381.31. Significantly increased pre- and post-procedure times in the hospital setting account for this difference. Although patient satisfaction was outside the scope of the current study, other studies have found time to be a major contributor to patient satisfaction [28-30]. As a result, we suspect the approximately 2.5 hours saved during the total visit time in the OBL setting would improve patient satisfaction. 

Several limitations to this study exist. First, costs associated with personnel, equipment, space, and materials, as well as procedural time estimates, vary widely across institutions, and costs may vary over time due to inflation. Nevertheless, the methodology and development of the TDABC models in this study are reproducible and can be used to model costs in other settings. Second, this study did not take into account other factors contributing to the choice of procedure setting, including differences in reimbursements, patient preference, or space availability. Opportunity costs may also affect the choice of treatment setting, as more resource-intensive procedures may limit additional procedures being performed in the same setting. Last, we did not consider other costs associated with pre-and post-procedure work-up, including office visits, laboratory tests, and imaging studies. However, these costs would be unchanged at our institution regardless of procedure setting.

## Conclusions

This TDABC analysis of PAE found that there is a modest decreased cost associated with procedures performed within an OBL compared to hospital settings. Moreover, the overall visit time is significantly lower within the OBL setting. Future studies aim to determine how other factors, such as patient satisfaction and reimbursement, may influence the choice of procedure setting.
